# The Polish version of the Process-Based Assessment Tool (PBAT)—The measure of processes of change in psychological interventions

**DOI:** 10.1371/journal.pone.0304661

**Published:** 2024-06-26

**Authors:** Maria Cyniak-Cieciura, Joanna Dudek, Paweł Ostaszewski

**Affiliations:** 1 Institute of Psychology, SWPS University, Advanced Clinical Studies and Therapy Excellence Centre, Warsaw, Poland; 2 Faculty of Psychology, SWPS University, Warsaw, Poland; 3 Institute of Psychology, SWPS University, Warsaw, Poland; Kitami Institute of Technology, JAPAN

## Abstract

The aim of this study was to conduct a Polish adaptation of Process-Based Assessment Tool (PBAT), to be used primarily for measurement of the processes of change occurring within psychological interventions, regardless of the therapeutic approach. PBAT includes a set of statements related to negative and positive behaviors in the domains of selection, variation, and retention, as it is theoretically embedded in the evolutionary approach. The tool’s construction was determined by resolving the issue of ergodic error, hence employs an idiographic approach. A total of 602 (319 F, 281 M) participants in the age 18–85 took part in the study. Apart from the original 21 PBAT items, two additional items related to self-care vs. self-impatience were tested. The included criterion variables related to the assessment of individual functioning in terms of distress (sadness, anxiety, stress, anger, lack of social support), health (health and vitality levels), the fulfillment or frustration of autonomy, connection, and competence need, as well as well-being (life-satisfaction and sense of professional burnout). The machine learning Boruta algorithm was utilized. PBAT items significantly predicted criterion variables. Positive selection behaviors were strongest predictors of Health, Vitality, Life-satisfaction as well as satisfaction of autonomy, connection and competence needs. Negative selection behaviors were strongest predictors of distress, lack of social support, work burnout as well as the frustration of autonomy, connection and competence needs. Overall, the PBAT items were more predictive of variables encompassing negative aspects of functioning than positive aspects or well-being. The overall relationships and conclusions are consistent with those obtained in the original study. The Polish version of PBAT is recommended for use in further scientific research and therapeutic processes.

## Introduction

The Process-Based Assessment Tool (PBAT) is primarily intended to be used for intensive and repeated measurement (ecological momentary assessment) of the processes of change occurring within various psychological interventions, regardless of the therapeutic approach employed [1] and in both clinical and non-clinical settings. The tool’s authors have spent years studying and analyzing research on the mediational processes involved in achieving change and personal goals within the psychotherapy process [2–4] of various problems (mostly clinical). This research direction was chosen by analyzing the widely discussed limitations of the current utility within the Diagnostic and Statistical Manual of Mental Disorders (DSM [5]) and the International Classification of Diseases (ICD, [6]) for clinical diagnosis of mental disorders [7,8], as well as the limitations of diagnosis-based therapy effectiveness [9–12]. Their research led them to develop a new approach to the treatment of mental disorders, called Process-Based Therapy (PBT; [13]). PBT describes the "contextually specific use of evidence-based processes linked to evidence-based procedures to help solve the problems and promote the prosperity of particular people" ([4], p. 38). Within PBT, processes of change are defined as "theory-based, dynamic, progressive, contextually bound, modifiable, and multilevel changes or mechanisms that occur in predictable, empirically established sequences oriented toward desirable outcomes" [1], p. 201). The processes of change are embedded in the Extended Evolutionary Meta-Model (EEMM; [2,14] which describes them in relation to four aspects of evolving systems [2]: variation, selection, retention, and context. In order to achieve individual goals, processes of change need to be profound and flexible (variation), allow for the assessment of their effectiveness for the pursued goals (selection), be sustainable over time (retention), and be context-sensitive to predict success or failure. Of particular importance is that embedding in the evolutionary model implies that these processes are not limited to psychopathology alone but are applicable to the entire population. They encompass behaviors exhibited by individuals that either contribute to general well-being and prosperity (the positive ones) or lead to difficulties (the negative ones), which may, in some instances, develop into symptoms commonly associated with psychopathology. The interventions focus on enhancing positive processes (positive behaviors) and reducing negative ones is anticipated to lead to the desired goals for individuals undergoing the intervention.

PBAT includes a pool of items (see Table 1) related to negative and positive processes—behaviors in the domains of selection (17 items), variation (2 items), and retention (2 items). Selection-related behaviors encompass aspects of affect, cognition, attention, self, motivation, and overt behavior. These statements were developed by a conceptually diverse team of experts in Acceptance and Commitment Therapy, Cognitive-Behavioral Therapy, Schema Therapy, Psychodynamic Therapy, Positive Psychology, therapy of adults, youth, and adolescents, social psychology, and methodology. The items focused on positive and negative behaviors related to the above-mentioned dimensions (variation, selection, and retention) of the EEMM and manifested in the areas of affect, cognition, attention, self, motivation, overt behavior, sociocultural, and biophysiological areas. They were strongly correlated with the moderators of therapy outcomes, as identified by Hayes et al. [15]. After approximately 10 revisions, the final item pool of 21 items was agreed upon and used in the original study.

**Table 1:** PBAT original and added items.

Process target	Negative behaviour	Positive behaviour
**Variation**	I felt stuck and unable to change my ineffective behavior (StuckUnableChange)	I was able to change my behavior, when changing helped my life (AbleToChangeBehaviour)
	I changed my environment, to improve my life (examples: removing temptation; reducing distractions; surrounding myself with positive influences) (ChangedEnvironment)	
**Selection**		
Affect/yearning to Feel	I did not find an appropriate outlet for my emotions (NoOutletForFeelings)	I was able to experience a range of emotions appropriate to the moment (ExperienceRangeEmotions)
Cognition/Yearning for Coherence	My thinking got in the way of things that were important to me (ThinkingGotInWay)	I used my thinking in ways that helped me live better(ThinkingHelpedLife)
Attention/Yearning to be Oriented	I struggled to connect with the moments in my day-to-day life(StruggleConnectMoments)	I paid attention to important things in my daily life (PaidAttToImportant)
Social Connection/Need for Connection	I did things that hurt my connection with people who are important to me(HurtConnections)	I did things to connect with people who are important to me (ConnectToPeople)
Motivation/Need for Autonomy	I did things only because I was complying with what others wanted me to do (Complying)	I chose to do things that were personally important to me (PersonalImport)
Overt Behaviour/Need for Competence	I did not find a meaningful way to challenge myself (NoMeaningfulChallenge)	I found personally important ways to challenge myself (ImportantChallenge)
Physical Health Behaviours	I acted in ways that hurt my physical health (HurtHealth)	I acted in ways that helped my physical health (HelpHealth)
Self-compassion*	I was intolerant and impatient with myself (SelfImpatience).	I showed myself care and tenderness when I needed it (Self-care).
**Retention**	I struggled to keep doing something that was good for me (StruggledToKeepDoing)	I stuck to strategies that seemed to have worked (StuckToStrategies)
		I stuck to what I cared about, even in the face of difficulties (StuckToWhatCared)
		I’ve used what I’ve learned in everyday life (UsedWhatLearned)

Note

*—additional items, not included in the original version; the abbreviations used within the manuscript are put in parentheses.

Then the final construction of the tool was determined by resolving the issue related to ergodic error, which assumes that for nomothetic measurement to translate to individuals, the measurement object must be stationary, and the same model must be invariant across subjects [16,17]–an assumption fundamentally unattainable when studying dynamic processes like processes of change. Hence, PBAT employs an idiographic approach to measure processes of change, which is reflected in its development methodology. Firstly, the tool is an item pool with each statement corresponding to one process of change and meant for an idionomic use. These statements are assessed separately on a wide-ranging 0–100 response scale. Secondly, due to the fact that traditional assumptions of classical test theory [18] do not apply to it, the verification of statements takes on a purely criterial character by relating each of them to the included criterion variables using, preliminary, correlations and ultimately the Boruta algorithm [19]. The included criterion variables relate to the assessment of individual functioning in terms of distress (perceived levels of sadness, anxiety, stress, anger, lack of social support), health (assessment of health and vitality levels), and the fulfillment or frustration of autonomy, connection, and competence needs (based on the self-determination theory by Ryan and Deci) [20].The validity of individual statements in the tool was based on the following assumptions: (1) positive items are moderately correlated with other positive items, negative items are moderately correlated with other negative items, and positive items are weakly correlated with negative items; (2) all items are linked to criterion variables in a theoretically coherent way (positive items predict strongly positive aspects of functioning, negative items predict strongly negative aspects of functioning).

The objective of our study is to conduct a Polish adaptation of PBAT, employing a similar approach to data analysis as in the original study. Additionally, we emphasize the necessity for further research utilizing the presented Polish version of the tool in a Polish context to confirm its validity and utility in intervention studies, especially among clinical populations.

In our study, we applied the same criterion variables as in the study of the original version of the tool, supplementing them with measures of well-being (life-satisfaction) and professional burnout. Our hypotheses reflect the expectations of the authors of the tool as follows: (1) positive items should moderately relate to other positive items, negative items should relate to other negative items, and positive items should exhibit weak correlations with negative items; (2) all items should relate to the chosen criterion in a manner where positive items are strong predictors of positive aspects of functioning, while negative items predict negative aspects of functioning. Failure to meet the second expectation should lead to the exclusion of the item from the final version of the tool.

Since the authors of the original version of PBAT [1] do not consider it a closed version and encourage the investigation of additional potential processes of change, we decided to test the functioning of two items (positive and negative) related to self-care vs. self-impatience. We developed these items based on research on compassion-focused psychotherapy [21], where the leading process of change appears to be a reduction in self-criticism and an increase in self-compassion [22], and its effectiveness has been partially confirmed in clinical studies [23,24]. Regarding these two items, we expected stronger associations between the self-care item and the other positive items, as well as between the self-impatience item and the other negative items. Regarding the two additional criterion variables, we expected that due to their generalized nature, their relationship with the items would be weaker compared to other criterion variables, but still significant. Finally, we anticipated that life-satisfaction would be more strongly predicted by positive items, while professional burnout would be predicted more by negative items.

## Materials and methods

### Procedure

The study was conducted online in June 2022. The participants were recruited by the professional research panel, the only exclusion criteria were being less than 18 years old. They were asked to fill in a set of self-reported measures (described below). All of the participants signed an informed consent. The study was conducted following the Declaration of Helsinki and received a positive opinion from the local Ethics Committee (No 6/2022).

### Participants

A total of 602 people completed the study: 319 women (53.0%) and 281 men (46.7%) in the age of 18–85 (*M* = 50.36, *SD* = 16.36). Most of them finished high school (*N* = 285, 47.3%), 29.0% had higher education (*N* = 175), 8.3% finished bachelor studies (*N* = 50) and 11.8% (*N* = 71) had vocational educational level. All other participants (*N* = 19, 3.2%) had a primary educational level. Most of the participants were living in the rural area (*N* = 238, 39.5%), 31.7% (*N* = 191) in a city with less than 100 000 citizens, 16.6% (*N* = 100) in a city with 100–500 000 residents, and 71 people (11.82%)—in a city with more than 500 000 citizens.

### Measures

The original 21 PBAT items and two additional items added based on consultation with the authors of original tool were translated to Polish independently by four translators, who are scientists and/or practitioners in third wave cognitive-behavioral approaches. The translations were then assessed by three competent judges (practitioners and scientists in the area of contextual behavioral science, behavioral analysis and psychodynamic approach. Within the expert panel developing original PBAT items specialists from different therapy modalities were engaged (see [1])). Choice of the final translation was based on the judges’ opinions. Following the ISPOR practices [25] back-translated items were checked by the authors of the original version of the tool and changed if needed. The original instruction and response scale were kept (see [1]), thus each item was rated on a scale from 0 to 100 (with 0 *= Strongly disagree*, *100 = Strongly agree*).

### Criterion-variables

The same measures were used to assess clinically relevant outcomes as well as need satisfaction and need frustration aspects as in the original study conducted by Ciarrochi et al [1]. Five STOP-D items were used to measure sadness, anxiety, stress, anger, and lack of social support [26]. A single item health measure [27] as well as the sum of points of three positive vitality items (“felt energized”, “vital and alive”, and “nearly always felt alert and awake”) from the vitality scale [28] were used. The same items measuring need satisfaction and frustration in the domains of autonomy (“I feel that my decisions reflect what I really want”; “I feel forced to do many things I wouldn’t choose to do”), competence (“I feel I can successfully complete difficult tasks”; “I feel insecure about my abilities”) and connection (‘I feel connected with people who care for me, and for whom I care”; “I feel the people who are important to me are cold and distant towards me”) were utilized (see [1,29]). More details can be found in Ciarrochi et al., [1] (the same ratings scales were used). All the items of the above-mentioned tools underwent the same translation procedure as the items of PBAT. Additional two outcome variables were added, that refer to the well-being aspect. Based on the findings of Cheung and Lucas [29] a single-item life satisfaction measure was used (“How satisfied are you with your life?”). As a single-item measures of work burnout were also found psychometrically sound [30,31], we used one to measure the level of burnout (“I felt burnt out from my work”). Both items were rated with a 0–100 response scale (0 –*Strongly Agree*, 100 –*Strongly disagree*). All PBAT items and criterion-variables measures were rated by participants in relation to the past week.

### Statistical analyses

To replicate the originally conducted analyses, firstly *r-Pearson* correlations and then a machine learning Boruta algorithm [19] was utilized across all subjects to estimate the importance of each item/attribute to relate to each of outcome variables. This method conducts a top-down exploration for items by assessing the significance of the original attributes in contrast to the significance attainable through randomly created shadow features of them. This is determined by evaluating their permuted duplicates and gradually removing non-essential features to establish a stable evaluation. In the initial step, shadow features are generated for all included items, which are uncorrelated with the responses. Subsequently, a random forest classifier is employed to compute Z-scores and evaluate the amount of information lost by excluding each item. The maximum Z-score from the shadow features is then determined and compared to the Z-scores of the items. If an item’s Z-score surpasses the maximum Z-score for the shadow feature, it is deemed a hit—an essential predictor of the criterion variable (where those with lower Z-scores are considered unimportant). This process is iterated to ensure its statistical robustness. In our study 99 iterations were performed. A confidence level of .01 was set. Items most consistently predictive across all iterations were retained; tentative items were finally confirmed if their median importance was higher than the median importance of maximal shadow attribute. The Bonferroni method was used to adjust for multiple comparisons. All the analyses were conducted with the use of *SPSS* v. 25, and *R* v. 4.3.1, package *Boruta* v. 8.0.0 [19,32].

### Results

Descriptive statistics of PBAT items and criterion variables are presented in Table 2. The normal distribution assumption for some of the PBAT items and criterion variables seem to be violated (skewness or kurtosis values exceed the range -1 - +1). Therefore non-parametric tests were used while performing correlation (*Kendall τ*) and comparison analyses (*U-Mann Whitney*).

**Table 2:** Descriptive statistics.

	*M*	*SD*	*Sk (SE)*	*Kt (SE)*
Positive selection behaviors				
PersonalImport	71.39	23.86	-.83 (.10)	.30 (.20)
HelpHealth	63.49	28.22	-.49 (.10)	-.74 (.20)
PaidAttToImportant	71.73	23.32	-.74 (.10)	-.07 (.20)
ConnectToPeople	62.09	29.18	-.54 (.10)	-.66 (.20)
ExperienceRangeEmotions	70.92	25.32	-.77 (.10)	-.08 (.20)
ImportantChallenge	54.86	27.89	-.21 (.10)	-.74 (.20)
ThinkingHelpedLife	63.96	27.12	-.58 (.10)	-.38 (.20)
UsedWhatLearned	76.38	23.22	-1.14 (.10)	.88 (.20)
StuckToWhatCared	66.52	26.14	-.65 (.10)	-.30 (.20)
SelfCare	56.01	29.41	-.20 (.10)	-.95 (.20)
Negative selection behaviors				
Complying	43.04	29.24	.13 (.10)	-1.07 (.20)
HurtHealth	32.93	28.98	.66 (.10)	-.69 (.20)
StruggleConnectMoments	31.97	29.25	.76 (.10)	-.48 (.20)
HurtConnect	33.27	31.05	.68 (.10)	-.74 (.20)
NoOutletForFeelings	34.79	30.30	.59 (.10)	-.78 (.20)
NoMeaningfulChallenge	45.80	30.06	.12 (.10)	-.95 (.20)
ThinkingGotInWay	39.24	31.36	.36 (.10)	-1.11 (.20)
ChangedEnvironment	46.41	30.81	.07 (.10)	-1.12 (.20)
SelfImpatience	36.08	30.76	.52 (.10)	-.92 (.20)
Variation				
StuckUnableToChange	35.05	30.39	.57 (.10)	-.81 (.20)
AbleToChangeBehavior	72.12	24.22	-.94 (.10)	.54 (.20)
Retention				
StruggledToKeepDoing	38.75	30.97	.41 (.10)	-1.03 (.20)
StuckToStrategies	62.49	25.55	-.47 (.10)	-.35 (.20)
Criterion variables				
Sadness	38.60	33.76	.41 (.10)	-1.23 (.20)
Anxiety	41.24	33.06	.32 (.10)	-1.26 (.20)
Stress	43.03	33.23	.25 (.10)	-1.31 (.20)
Anger	37.67	31.00	.51 (.10)	-.95 (.20)
NoSupp	36.98	32.66	.46 (.10)	-1.07 (.20)
Health	60.90	23.59	-.69 (.10)	-.07 (.20)
LS	61.37	28.91	-.65 (.10)	-.56 (.20)
WorkBurn	36.33	33.03	.53 (.10)	-1.03 (.20)
Vitality	148.15	70.33	-.05 (.10)	-.51 (.20)
AutS	131.68	45.91	-.53 (.10)	-.03 (.20)
AutF	70.60	54.59	.42 (.10)	-.77 (.20)
ConS	144.92	49.94	-.90 (.10)	.22 (.20)
ConF	57.71	53.04	.71 (.10)	-.49 (.20)
ComS	138.53	47.06	-.83 (.10)	.29 (.20)
ComF	57.67	53.08	.79 (.10)	-.30 (.20)

*Note*: *M*–mean, *SD*–standard deviation, *Sk–*skewness, *Kt–*kurtosis, *SE–*standard error; Sadness–the level of sadness; anxiety–the level of anxiety, Stress–the level of stress; Anger–the level of anger; NoSupp–feeling of lack of social support; Health–subjective assessment of the level of health; LS–life-satisfaction level; WorkBurn–the level of professional burnout; Vitality–the level of vitality; AutS–satisfaction of autonomy need; AutF–frustration of autonomy need; ConS–satisfaction of connection need; ConF–frustration of connection need; ComS–satisfaction of competence need; ComF–frustration of competence need; the abbreviations for PBAT items are described in Table 1.

Among positive selection behaviors, participants reported that they were most successful in leveraging their previous experience, while they were least successful in setting themselves significant personal challenges. Among negative selection behaviors, focusing on the present moment proved to be the most difficult, while changing their own environment posed the least difficulty.

Significant sex differences were observed in relation to five PBAT items. Women indicated that they find it easier to experience a variety of different emotions in response to situations, have more hindering thoughts, find it harder to focus on the present moment, and comply more easily. On the other hand, men pointed out that they tend to adhere more frequently to strategies that seem to work. Regarding the criterion variables, significant sex differences were observed in the levels of perceived sadness, anxiety, stress, anger, frustration in autonomy need, and satisfaction in interpersonal relationships need (higher scores among women), as well as in the sense of vitality (higher score among men).

The PBAT items were significantly interrelated (see Table 3). Generally, most positive behaviors were positively intercorrelated with other positive behaviors, and negative behaviors–with other negatives. At the same time negative relationships were weak and found for intercorrelations between positive and negative behaviors.

**Table 3:** PBAT items interrelationships as well as relationships between PBAT items and criterion variables.

	1		2	3	4	5	6	7	8	9	10	11	12	13	14	15	16	17	18	19	20	21	22	23
Positive selection behaviors																								
1. PersonalImport																								
2. HelpHealth	.303**																							
3. PaidAttToImportant	.416**		.350**																					
4. ConnectToPeople	.433**		.194**	.285**																				
5. ExperienceRangeEmotions	.194**		.129**	.165**	.196**																			
6. ImportantChallenge	.372**		.282**	.356**	.323**	.132**																		
7. ThinkingHelpedLife	.447**		.346**	.420**	.278**	.093**	.408**																	
8. UsedWhatLearned	.382**		.281**	.405**	.251**	.164**	.276**	.385**																
9. StuckToWhatCared	.442**		.278**	.424**	.342**	.191**	.332**	.395**	.454**															
10. SelfCare	.358**		.291**	.294**	.255**	.142**	.351**	.374**	.327**	.331**														
Negative selection behaviors																								
11. Complying	-.025		-.097**	-.078**	.056*	.067*	.030	-.070*	-.110**	-.060*	-.024													
12. HurtHealth	-.116**		-.279**	-.217**	-.023	.011	-.068*	-.158**	-.210**	-.121**	-.119**	.301**												
13. StruggleConnectMoments	-.123**		-.149**	-.195**	-.027	.004	-.083**	-.178**	-.238**	-.126**	-.058*	.334**	.402**											
14. HurtConnect	-.051		-.109**	-.161**	.041	.125**	.018	-.074**	-.139**	-.047	-.035	.304**	.369**	.340**										
15. NoOutletForFeelings	-.113**		-.181**	-.180**	-.027	.030	-.097**	-.176**	-.225**	-.109**	-.122**	.344**	.430**	.471**	.344**									
16. NoMeaningfulChallenge	-.038		-.078**	-.108**	-.022	.059*	-.088**	-.085**	-.093**	-.065*	-.075**	.177**	.242**	.231**	.163**	.278**								
17. ThinkingGotInWay	-.136**		-.150**	-.193**	-.002	.067*	-.108**	-.183**	-.190**	-.114**	-.088**	.329**	.392**	.505**	.339**	.485**	.263**							
18. ChangedEnvironment	.221**		.179**	.217**	.238**	.127**	.252**	.239**	.158**	.292**	.223**	.039	.033	.063*	.088**	.053	-.030	.053						
19. SelfImpatience	-.121**		-.172**	-.153**	-.035	.028	-.084**	-.173**	-.194**	-.091**	-.157**	.320**	.389**	.448**	.334**	.454**	.238**	.414**	.118**					
Variation																								
20. AbleToChangeBehavior		,283**	,236**	,254**	,264**	,263**	,292**	,300**	,243**	,222**	,232**	,031	-,033	-,052	,057*	-,089**	,015	-,041	,187**	-,022				
21.StuckUnableToChange		-,125**	-,142**	-,185**	-,006	,050	-,086**	-,185**	-,210**	-,107**	-,136**	,325**	,405**	,529**	,374**	,473**	,271**	,499**	,054	,425**	-,037			
Retention																								
22. StuckToStrategies	.344**		.220**	.320**	.263**	.196**	.377**	.375**	.317**	.333**	.262**	.042	-.044	-.090**	.019	-.030	.044	-.069*	.157**	-.109**	.251**	-.074**		
23. StruggledToKeepDoing	-.107**		-.164**	-.209**	-.005	.091**	-.085**	-.153**	-.200**	-.113**	-.070*	.316**	.430**	.475**	.393**	.429**	.253**	.456**	.051	.353**	-.006	.481**	-.025	
Criterion variables																								
Sadness	-.121**		-.151**	-.102**	-.079**	.038	-.131**	-.143**	-.118**	-.060*	-.101**	.251**	.275**	.399**	.211**	.411**	.172**	.386**	.035	.329**	-.036	.407**	-.068*	.321**
Anxiety	-.094**		-.159**	-.095**	-.058*	.079**	-.147**	-.148**	-.112**	-.045	-.105**	.251**	.298**	.376**	.218**	.390**	.115**	.370**	.024	.332**	-.057*	.386**	-.071*	.317**
Stress	-.074**		-.135**	-.074**	-.035	.087**	-.102**	-.148**	-.104**	-.019	-.077**	.260**	.265**	.372**	.218**	.388**	.118**	.370**	.057*	.337**	-.057*	.379**	-.037	.302**
Anger	-.110**		-.132**	-.097**	-.030	.044	-.083**	-.123**	-.106**	-.053	-.102**	.236**	.267**	.344**	.296**	.372**	.102**	.318**	.048	.347**	-.048	.358**	-.033	.287**
NoSupp	-.110**		-.144**	-.096**	-.055	.029	-.069*	-.095**	-.135**	-.042	-.104**	.238**	.260**	.349**	.259**	.357**	.172**	.336**	.072*	.361**	-.073*	.364**	-.035	.313**
Health	.098**		.178**	.114**	.177**	.079**	.139**	.126**	.087**	.074**	.172**	-.067*	-.169**	-.095**	-.045	-.107**	-.040	-.088**	.101**	-.117**	.117**	-.118**	.114**	-.112**
LS	.185**		.207**	.217**	.154**	.050	.200**	.204**	.188**	.162**	.207**	-.094**	-.117**	-.125**	-.075*	-.147**	-.030	-.138**	.099**	-.092**	.185**	-.156**	.159**	-.121**
WorkBurn	-.068*		-.087**	-.079**	-.018	.052	-.072*	-.095**	-.134**	-.050	-.050	.230**	.174**	.282**	.202**	.301**	.181**	.295**	.098**	.241**	-.052	.260**	-.020	.251**
Vitality	.233**		.277**	.204**	.256**	.053	.320**	.260**	.219**	.181**	.265**	-.033	-.132**	-.134**	-.013	-.168**	-.082**	-.145**	.165**	-.146**	.190**	-.188**	.204**	-.120**
AutS	.387**		.300**	.387**	.250**	.156**	.328**	.368**	.395**	.361**	.367**	-.111**	-.217**	-.211**	-.134**	-.257**	-.125**	-.238**	.193**	-.228**	.240**	-.261**	.308**	-.216**
AutF	-.079**		-.112**	-.065*	.015	.070*	-.005	-.110**	-.127**	-.064*	-.073**	.348**	.252**	.319**	.264**	.319**	.147**	.355**	.092**	.300**	.033	.353**	-.021	.282**
ConS	.306**		.277**	.338**	.282**	.094**	.227**	.281**	.319**	.286**	.243**	-.115**	-.212**	-.218**	-.197**	-.234**	-.098**	-.184**	.111**	-.179**	.219**	-.242**	.193**	-.176**
ConF	-.127**		-.102**	-.151**	-.049	.026	-.076**	-.130**	-.159**	-.097**	-.089**	.259**	.291**	.364**	.323**	.367**	.154**	.337**	.061*	.327**	-.045	.393**	-.069*	.281**
ComS	.333**		.309**	.357**	.233**	.093**	.315**	.346**	.407**	.306**	.285**	-.126**	-.203**	-.255**	-.123**	-.257**	-.109**	-.235**	.119**	-.228**	.220**	-.261**	.252**	-.234**
ComF	-.164**		-.146**	-.177**	-.075**	-.027	-.135**	-.197**	-.240**	-.126**	-.140**	.235**	.270**	.384**	.230**	.345**	.207**	.344**	.042	.368**	-.090**	.388**	-.121**	.352**

Note

*—*p <* .05

**—p < .001; Sadness–the level of sadness; anxiety–the level of anxiety, Stress–the level of stress; Anger–the level of anger; NoSupp–feeling of lack of social support; Health–subjective assessment of the level of health; LS–life-satisfaction level; WorkBurn–the level of professional burnout; Vitality–the level of vitality; AutS–satisfaction of autonomy need; AutF–frustration of autonomy need; ConS–satisfaction of connection need; ConF–frustration of connection need; ComS–satisfaction of competence need; ComF–frustration of competence need; the abbreviations for PBAT items are described in Table 1.

There were plenty of significant relationships between PBAT items and criterion variables (Table 3). The strongest positive relationships were found between:

- engaging in disturbing thoughts, feeling stuck and unable to change, struggling to connect with present moment as well as finding no proper outlet for one’s feelings and sadness, anxiety, stress, anger, lack of support, work burnout, connection and competence needs frustration;
- engaging in helping health behaviors, paying attention to important things, as well as self-care and health, life-satisfaction, competence satisfaction need.

The individual PBAT items were found to significantly predict the criterion variables (Table 4). Positive selection behaviors were strongest predictors of Health, Vitality, Life-satisfaction as well as satisfaction of autonomy, connection and competence needs. At the same time, negative selection behaviors were strongest predictors of distress (sadness, anxiety, stress, anger), lack of social support, work burnout as well as the frustration of autonomy, connection and competence needs. One item each regarding variation and retention were significant predictors of all or nearly all of the criterion variables included in the study (feeling stuck and unable to change, and struggling to keep doing important things, respectively). Overall, the PBAT items were more predictive of variables encompassing negative aspects of functioning (unpleasant emotions, lack of social support, occupational burnout, frustration of needs) than positive aspects or well-being. The top predictor of all negative states was “found no appropriate outlet for feelings”; the top predictor of positive aspects was “connected with important people”. Two added items of self-care and self-impatience were significant and important predictors of 12 and 14 criterion variables respectively. Three items excluded in the study of Ciarrochi et al. [1] were also significant and important predictors to at least 7 criterion variables. Three PBAT items: “Found no meaningful challenge”, “able to change behavior, when changing helped”, and “stuck to strategies that worked” were less predictive comparing to other items, as they predicted only 4 criterion variables each. At the same time, their importance was quite high, and to support PBAT content validity we do not recommend to exclude them from the Polish version of PBAT. Detailed information regarding the significant predictors of individual criterion variables is presented in Table 4.

**Table 4:** Importance of PBAT items for criterion variables.

	Sadness	Anxiety	Stress	Anger	NoSupp	Health	LS	WorkBurn	Vitality	AutS	AutF	ConS	ConF	ComS	ComF
Positive selection behaviors															
PersonalImport	**3.21**	1.54	0.94	**2.91**	1.43	1.64	2.16	1.34	**6.57**	**11.22**	**2.69**	**8.31**	**2.52**	**9.32**	1.63
HelpHealth	**3.40**	**4.60**	1.99	**2.98**	**4.36**	**8.27**	**3.18**	1.25	**12.62**	**6.19**	2.20	**6.87**	-0.70	**10.85**	1.06
PaidAttToImportant	1.77	1.25	1.05	**3.00**	1.08	0.45	**8.81**	-0.14	**3.17**	**10.36**	**2.68**	**10.51**	1.73	**8.94**	2.44
ConnectToPeople	**4.44**	**2.75**	**2.80**	**2.53**	1.78	**8.84**	1.16	**2.73**	**9.51**	**2.98**	**3.38**	**11.48**	**2.79**	**4.92**	**2.95**
ExperienceRangeEmotions	0.49	1.48	**2.48**	1.27	1.09	1.79	0.59	0.15	1.23	**2.64**	**2.67**	**2.31**	1.81	0.91	1.56
ImportantChallenge	**4.92**	**5.07**	**3.63**	1.44	2.30	**2.68**	**2.63**	1.21	**13.52**	**8.55**	**3.84**	**2.90**	1.41	**7.98**	2.32
ThinkingHelpedLife	**2.94**	**2.96**	**3.04**	**2.67**	0.58	1.06	**2.86**	1.34	**6.57**	**7.72**	**3.88**	**5.77**	**3.52**	**8.68**	**4.68**
UsedWhatLearned	1.41	**3.89**	1.47	2.47	2.06	1.40	**4.84**	**4.87**	**7.42**	**11.05**	**3.08**	**7.15**	**2.86**	**17.93**	**7.53**
StuckToWhatCared	1.09	2.05	2.30	0.48	**2.62**	1.19	**3.18**	2.20	2.19	**8.55**	**2.84**	**5.95**	2.18	**6.27**	**2.47**
SelfCare	1.24	**3.32**	1.33	**2.78**	**3.13**	**5.67**	**6.31**	1.29	**7.83**	**14.20**	**2.75**	**4.18**	**2.49**	**8.01**	**2.39**
Negative selection behaviors															
Complying	**7.99**	**7.50**	**7.01**	**4.42**	**6.76**	2.16	**3.25**	**9.88**	1.43	1.78	**16.79**	**3.36**	**6.93**	0.84	**2.86**
HurtHealth	**7.57**	**9.64**	**6.54**	**6.80**	**5.80**	**8.98**	2.15	1.67	**2.68**	**6.10**	**7.25**	**4.34**	**5.19**	**3.42**	**3.26**
StruggleConnectMoments	**19.79**	**18.45**	**18.26**	**14.06**	**13.32**	**3.41**	0.88	**8.92**	**4.57**	**4.23**	**8.04**	**5.99**	**10.76**	**7.27**	**13.88**
HurtConnect	**4.04**	**4.16**	**4.28**	**11.69**	**7.54**	**3.56**	0.55	**5.34**	2.31	**3.15**	**7.12**	**6.61**	**14.79**	1.56	**3.46**
NoOutletForFeelings	**24.60**	**23.47**	**22.33**	**20.50**	**17.28**	**3.95**	**2.43**	**12.76**	**5.74**	**9.79**	**10.43**	**10.68**	**16.05**	**8.24**	**9.45**
NoMeaningfulChange	1.69	0.12	1.61	0.65	**3.65**	-0.14	-0.64	**3.11**	**3.46**	1.60	1.88	0.61	0.51	0.59	**6.49**
ThinkingGotInWay	**18.32**	**16.24**	**15.39**	**7.91**	**13.10**	**4.24**	1.34	**11.10**	**4.22**	**5.94**	**14.69**	**3.61**	**11.37**	**5.60**	**10.07**
ChangedEnvironment	1.23	0.99	1.52	**3.72**	**3.88**	**5.46**	**3.22**	**4.99**	**2.98**	**3.25**	**5.76**	0.31	1.62	**3.46**	**2.55**
SelfImpatience	**12.38**	**13.78**	**15.32**	**14.17**	**13.43**	**4.94**	-0.11	**7.21**	**3.41**	**5.26**	**9.40**	**2.43**	**9.67**	**6.41**	**12.16**
Variation															
StuckUnableToChange	**21.61**	**19.49**	**19.63**	**15.40**	**18.08**	**5.67**	**3.35**	**9.19**	**9.06**	**8.83**	**14.27**	**8.78**	**17.14**	**8.35**	**12.79**
AbleToChangeBehavior	0.80	-0.42	1.65	0.76	1.88	**3.23**	**4.33**	1.65	2.26	1.64	1.70	**5.34**	1.61	**3.15**	1.88
Retention															
StruggledToKeepDoing	**11.40**	**10.44**	**9.15**	**7.28**	**10.27**	**4.81**	**2.97**	**5.70**	**5.15**	**6.34**	**3.22**	**4.07**	1.34	**4.55**	**12.47**
StuckToStrategies	1.01	1.96	1.37	0.68	1.52	1.70	**3.81**	1.08	2.40	**6.29**	2.13	**2.92**	1.62	**3.95**	1.81

*Note*: the importance values calculated based on the Boruta algorithm; Sadness–the level of sadness; anxiety–the level of anxiety, Stress–the level of stress; Anger–the level of anger; NoSupp–feeling of lack of social support; Health–subjective assessment of the level of health; LS–life-satisfaction level; WorkBurn–the level of professional burnout; Vitality–the level of vitality; AutS–satisfaction of autonomy need; AutF–frustration of autonomy need; ConS–satisfaction of connection need; ConF–frustration of connection need; ComS–satisfaction of competence need; ComF–frustration of competence need; the abbreviations for PBAT items are described in Table 1.

## Discussion

The described study aimed to do a Polish adaptation of a tool used to measure specific behaviors—the processes of change in psychological condition and psychological interventions. The obtained results allow us to consider this goal as achieved. The study applied the same methodological approach and included the same criterion variables as in the study on the original version of the tool [1]. The tool itself was enriched with two additional items related to self-care and expressing impatience towards self (based on the compassion focused therapy approach, see [21]), as well as two additional criterion variables: life-satisfaction and a sense of work burnout. Thus, in addition to clinical variables and those related to satisfaction or frustration of needs, the aspects of well-being were also taken into account.

Although individual items were assessed by participants differently compared to the study by Ciarrochi et al. [1], the overall relationships and conclusions are consistent with the results obtained by the authors of the original version of the tool and support set hypotheses. This allows us to recommend the Polish version of PBAT for use in further scientific research and therapeutic processes.

The PBAT items were mutually correlated with each other in direction and strength consistent with expectations. Items related to positive selection behaviors were most strongly associated with other items in that group, and items related to negative selection behaviors were most strongly associated with other items related to negative behaviors. The relationships between positive selection behaviors and negative selection behaviors were weaker but significant. Correlations with criterion variables were consistent with those obtained in the study of the original version and aligned with expectations. Items capturing positive aspects of behavior were generally most strongly correlated with variables encompassing positive aspects of functioning (namely Health, Vitality, Life-satisfaction, and satisfaction of autonomy, connection, and competence needs). Items capturing negative aspects of behavior were generally most strongly correlated with variables encompassing negative aspects of functioning (distress, lack of social support, a sense of work burnout, and frustration of the aforementioned needs).

Analysis using the Boruta algorithm [19] allowed for determining the predictive value of individual PBAT items with respect to the included criterion variables. Similar to the correlations, values for individual PBAT items differed compared to the results obtained by Ciarrochi et al. [1], but the overall conclusions are consistent and similar.

Positive selection behaviors were the strongest predictors of criterion variables that described positive aspects of people’s functioning, while negative selection behaviors were the strongest predictors of negative aspects. Overall, PBAT items were more predictive of variables encompassing negative aspects of functioning (distress, lack of social support, professional burnout, frustration of needs) than positive aspects or well-being. More general variables, encompassing a broad range of functioning and including aspects detailed by other variables (namely life-satisfaction and level of professional burnout), were slightly but still significantly predicted by PBAT items, as expected.

The least effective PBAT items (namely: ‘Able to experience a range of emotions appropriate to the moment’, ’Found no meaningful challenge’, ’Able to change behavior when changing helped’, and ’Stuck to strategies that worked’) predicted only four criterion variables each, doing so to a weak or moderate degree. These were different items than in the study by Ciarrochi et al. [1]. Experiencing range of different emotions refers to the acceptance domain from the acceptance and commitment therapy’s model for psychological flexibility [33]. We have found difficulty with functioning of the items from this domain in previous Polish adaptations of psychological flexibility measures (unpublished yet) and suggested that there are possibly significant cultural differences in understanding the affect domain. Similar problems were noticed by Trinidade et al. [34], Suganda and Abidin [35] as well as Tabrizi et al. [36] in their studies on psychological flexibility measures. Also, the lower importance of the rest three items while compared to the original study suggest some cultural sensitivity of PBAT items. However, as two items: ’Able to change behavior when changing helped’, and ’Stuck to strategies that worked’ are the only representatives of positive variation and retention behaviours, removing them would be detrimental to content validity of the tool. Therefore we recommend to include these items in the Polish version.

Referring to the three items removed from the original version of the tool, namely the leveraging their previous experience, the attachment to what is cared (positive selective behaviors), and making specific changes in one’s environment (negative selective behavior), in our study, the first two processes were found to be strongly and significantly associated with satisfaction of all three examined needs. Therefore, we see no grounds for their removal from the PBAT item pool. Some controversies arise regarding the last item—making changes in the environment, which emerged as a significant and relatively strong predictor for frustration of the need for autonomy and occupational burnout. Simultaneously, it was also a comparably strong predictor of self-rated health. Hence, in the Polish context, this process seems to function differently than in the American sample, and due to the lack of theoretical consistency in the obtained results, we recommend removing this item from the PBAT item pool. The addition of two extra items related to self-care and expressing self-impatience proved to significantly predict at least 12 out of the 15 included criterion variables, justifying their inclusion in the tool.

Sadness, anxiety and stress were best predicted by finding no appropriate outlet for one’s feelings, feeling stuck and unable to change, as well as by struggling to connect with present moment. Anger and lack of social support was best predicted by finding no appropriate outlet for one’s feelings, feeling stuck and unable to change, as well as by self-impatience. The sense of work burnout was best predicted by finding no appropriate outlet for one’s feelings, difficult thoughts, and complying. Health was best predicted by the hurting and helping health behaviors, as well as by connecting to important people. Vitality was best predicted by setting important challenges, engaging in helping health behaviors, as well as connecting to important people. Life-satisfaction was best predicted by paying attention to important things, self-care behaviors, and using previous experience.

Considering the needs, satisfaction of autonomy was best predicted by self-care behaviors, engagement in personally important things, and the use of previous experience. Frustration of autonomy was best predicted by complying, difficult thoughts, and feeling stuck and unable to change. Satisfaction of connection was best predicted by connecting to important people, paying attention to important things and founding no proper outlet for feelings. The frustration of connection was best predicted by feeling stuck and unable to change, hurting social connections, and founding no proper outlet for feelings. Finally, satisfaction of competence was best predicted by using previous experience, helping one’s health, doing personally important things, and frustration of competence–by struggling to keep doing important things, feeling stuck and unable to change, as well as by being self-impatient.

Our study suggests that, within a Polish context, while individual PBAT items exhibited some cultural specificity, the tool as a whole functions similarly to its original version. This supports its use in further studies among Polish samples, as well as its application in cross-cultural studies examining the nature of change processes, thus expanding the potential global utility of PBAT. To the best of our knowledge, this is the first Polish study directly addressing processes of change. Its results demonstrate a clear connection between these processes and various aspects of psychological functioning, encompassing affect, social connections, physical health, and overall well-being.

The limitations of this study primarily pertain to the limited, non-representative sample of individuals who participated in the study and its cross-sectional nature. As the study primarily aimed at replication, the methodology of the original study was adopted, including the selection of a non-clinical sample. Indeed, limiting the use of the tool solely to clinical populations could potentially skew the results, accentuating the connections between negative processes and criterion variables. Such an approach would be ill-advised during the tool’s development or adaptation phase. However, future research should involve clinical samples and incorporate longitudinal studies, especially within groups undergoing interventions targeting specific change processes. This would further solidify the tool’s validity in measuring changes occurring within psychological interventions.

## Supporting information

S1 File (DOCX)
