# Supplementary material for: The Polish version of the Process-Based Assessment Tool (PBAT)—The measure of processes of change in psychological interventions
Source: PLoS One. 2024 Jun 26;19(6):e0304661. doi: 10.1371/journal.pone.0304661 (PMC11207177; doi:10.1371/journal.pone.0304661)
Supplement: S1 File — (DOCX) [file pone.0304661.s001.docx]

**Supplementary materials**

Table A

Sex differences for PBAT items and criterion variables.

| Variables | F | | M | | *U* |
| --- | --- | --- | --- | --- | --- |
|  | *M* | *SD* | *M* | *SD* |  |
| AbleToChangeBehavior | 71.90 | 23.81 | 72.48 | 24.75 | 43354.50 |
| HurtConnect | 32.57 | 31.89 | 33.90 | 30.14 | 41596.50 |
| ExperienceRangeEmotions | 74.02 | 25.79 | 67.59 | 24.27 | 36781.50** |
| StruggledToKeepDoing | 40.37 | 32.23 | 36.70 | 29.44 | 41371.50 |
| NoMeaningfulChange | 44.42 | 30.79 | 47.23 | 29.23 | 40646.00 |
| HelpHealth | 64.18 | 28.52 | 62.77 | 27.98 | 42642.50 |
| ThinkingGotInWay | 43.07 | 33.28 | 34.72 | 28.49 | 37777.50* |
| PaidAddToImportant | 72.03 | 23.92 | 71.56 | 22.53 | 42749.00 |
| Complying | 45.29 | 30.50 | 40.27 | 27.54 | 39461.50* |
| StuckToStrategies | 59.83 | 26.43 | 65.49 | 24.20 | 38862.50* |
| ImportantChallenge | 52.84 | 29.04 | 57.35 | 26.35 | 39645.00 |
| StuckUnableToChange | 37.51 | 32.00 | 32.17 | 28.32 | 39220.50 |
| ThinkingHelpedLife | 62.63 | 28.03 | 65.75 | 25.87 | 41448.00 |
| StruggleConnectMoments | 35.08 | 31.40 | 28.15 | 26.01 | 37911.50* |
| ConnectToPeople | 61.29 | 29.80 | 62.86 | 28.55 | 42815.00 |
| PersonalImport | 70.10 | 25.22 | 72.81 | 22.26 | 42007.50 |
| HurtHealth | 32.02 | 29.44 | 33.76 | 28.47 | 40901.50 |
| NoOutletForFeelings | 36.04 | 31.87 | 33.12 | 28.34 | 42002.50 |
| ChangedEnvironment | 47.09 | 31.29 | 45.77 | 30.32 | 42275.00 |
| StuckToWhatCared | 65.81 | 26.11 | 67.36 | 26.29 | 42646.00 |
| UsedWhatLearned | 75.75 | 23.19 | 77.49 | 22.80 | 40002.50 |
| SelfCare | 56.11 | 30.25 | 56.21 | 28.33 | 43888.00 |
| SelfUnpatience | 38.12 | 32.66 | 33.37 | 28.06 | 40351.00 |
| Sad | 43.07 | 35.12 | 33.13 | 31.24 | 36384.00** |
| Anxiety | 48.48 | 34.51 | 32.69 | 29.08 | 32076.50** |
| Stress | 49.22 | 35.28 | 35.60 | 29.03 | 33277.00** |
| Angry | 40.77 | 33.13 | 33.98 | 28.03 | 38624.00* |
| NoSupport | 38.67 | 34.01 | 34.75 | 30.90 | 39393.50 |
| Health | 60.80 | 23.25 | 61.21 | 23.95 | 43467.50 |
| LifeSatisfaction | 60.64 | 28.23 | 62.09 | 29.69 | 36083.00 |
| WorkBurnout | 38.71 | 34.35 | 33.31 | 31.18 | 39575.00 |
| Vitality | 140.80 | 71.13 | 157.05 | 68.39 | 37926.50* |
| AutonomySatisfaction | 131.44 | 46.79 | 132.54 | 44.55 | 43590.00 |
| AutonomyFrustration | 76.65 | 56.49 | 62.96 | 50.97 | 36603.00* |
| ConnectionSatisfaction | 148.84 | 49.48 | 140.89 | 49.82 | 39308.50* |
| ConnectionFrustration | 58.92 | 55.42 | 55.92 | 49.91 | 41229.50 |
| CompetenceSatisfaction | 137.34 | 49.12 | 140.55 | 43.96 | 43169.00 |
| CompetenceFrustration | 59.44 | 54.19 | 55.00 | 51.36 | 39151.00 |

**Appendix A**

**PBAT wersja polska**

Maria Cyniak-Cieciura*, Joanna Dudek, Paweł Ostaszewski

Zaznacz na skali od 0 do 100, w jakim stopniu zgadzasz się z każdym z poniższych stwierdzeń. Swoje odpowiedzi oprzyj na tym, jak zachowywałeś/aś się w ostatnim tygodniu/jak zachowujesz się w danym momencie. Nie ma poprawnych bądź błędnych odpowiedzi na poniższe stwierdzenia.

| Byłem/am w stanie zmienić swoje zachowanie, jeżeli było to dla mnie pomocne w życiu. |
| --- |
| 0 -------------------------------------------------------------------------------------------------------- 100 |
| Robiłem/am rzeczy, które psuły moją więź z innymi, ważnymi dla mnie ludźmi. |
| 0 -------------------------------------------------------------------------------------------------------- 100 |
| Byłem/am w stanie doświadczać wielu różnych emocji, adekwatnie do sytuacji. |
| 0 -------------------------------------------------------------------------------------------------------- 100 |
| Miałem/am trudność z robieniem tego, co jest dla mnie dobre. |
| 0 -------------------------------------------------------------------------------------------------------- 100 |
| Nie wymyśliłem/am, jak w wartościowy dla mnie sposób rzucić sobie wyzwanie. |
| 0 -------------------------------------------------------------------------------------------------------- 100 |
| Postępowałem/am w sposób, który sprzyjał mojemu zdrowiu fizycznemu. |
| 0 -------------------------------------------------------------------------------------------------------- 100 |
| Moje myśli przeszkadzały mi w robieniu tego, co dla mnie ważne. |
| 0 -------------------------------------------------------------------------------------------------------- 100 |
| W moim codziennym życiu poświęcałem/am uwagę rzeczom ważnym. |
| 0 -------------------------------------------------------------------------------------------------------- 100 |
| Robiłem/am coś tylko dlatego, że dostosowywałem się do tego, czego chcieli ode mnie inni. |
| 0 -------------------------------------------------------------------------------------------------------- 100 |
| Trzymałem/am się strategii, które wydawały się działać. |
| 0 -------------------------------------------------------------------------------------------------------- 100 |
| Odkryłem/am, w jaki sposób postawić przed sobą wyzwanie tak, aby było to dla mnie osobiście ważne. |
| 0 -------------------------------------------------------------------------------------------------------- 100 |
| Czułem/am się jak w potrzasku i niezdolny/a do zmiany mojego bezowocnego zachowania. |
| 0 -------------------------------------------------------------------------------------------------------- 100 |
| Używałem/am swoich myśli w taki sposób, aby pomagały mi lepiej żyć. |
| 0 -------------------------------------------------------------------------------------------------------- 100 |
| Na co dzień trudno było mi skupić się na tym, co działo się tu i teraz. |
| 0 -------------------------------------------------------------------------------------------------------- 100 |
| Podejmowałem/am działania, by zbliżyć się do osób, które są dla mnie ważne. |
| 0 -------------------------------------------------------------------------------------------------------- 100 |
| Wybierałem/am działania, które były dla mnie osobiście ważne. |
| 0 -------------------------------------------------------------------------------------------------------- 100 |
| Działałem/am w sposób, który szkodził mojemu zdrowiu fizycznemu. |
| 0 -------------------------------------------------------------------------------------------------------- 100 |
| Nie udawało mi się znaleźć odpowiedniego ujścia dla moich emocji. |
| 0 -------------------------------------------------------------------------------------------------------- 100 |
| Trzymałem/am się tego, na czym mi zależało, nawet w obliczu trudności. |
| 0 -------------------------------------------------------------------------------------------------------- 100 |
| Wykorzystywałem w życiu codziennym to, czego się nauczyłem. |
| 0 -------------------------------------------------------------------------------------------------------- 100 |
| Okazywałem/am sobie troskę i czułość, gdy tego potrzebowałem/am. |
| 0 -------------------------------------------------------------------------------------------------------- 100 |
| Byłem/am wobec siebie nietolerancyjna/y i niecierpliwy/a. |
| 0 -------------------------------------------------------------------------------------------------------- 100 |
